# Parenteral neonatal priming followed by heterologous mucosal booster favors IgM^+^ memory B cell induction over systemic plasma cell differentiation

**DOI:** 10.3389/fimmu.2026.1792155

**Published:** 2026-05-05

**Authors:** Poorya Foroutan Pajoohian, Audur Anna Aradottir Pind, Jenny Lorena Molina Estupiñan, Dennis Christensen, Gabriel Kristian Pedersen, Thorunn A. Olafsdottir, Ingileif Jonsdottir, Stefania P. Bjarnarson

**Affiliations:** 1 Faculty of Medicine, Biomedical Center, School of Health Sciences, University of Iceland, Reykjavik, Iceland; 2 Department of Immunology, Landspitali, The National University Hospital of Iceland, Reykjavik, Iceland; 3 Center for Vaccine Research, Statens Serum Institut, Copenhagen, Denmark

**Keywords:** germinal center, memory B cell, neonatal immunization, plasma cells, prime-boost immunization

## Abstract

Early-life vaccinations often yield short-lived antibody (Ab) responses due to limited germinal center (GC) reaction and plasma cell (PC) survival. The aim of the study was to assess how vaccine delivery routes (homologous versus heterologous) in mice shape early-life GC dynamics and outputs. 14 days post-booster immunization, spleen, cervical (CLN), and inguinal (ILN) lymph nodes were analyzed for GC B cells, T follicular helper (T_FH_) and regulatory (T_FR_) cells, and GC-derived memory B cells and expression of B cell activating factor receptor (BAFF-R)/transmembrane activator and cyclophilin ligand interactor (TACI) on GC and memory B cells. Plasmablast/PC and their B cell maturation antigen (BCMA) expression, vaccine-specific Ab-secreting cells (ASCs), and serum and salivary Abs were also assessed. We observed that the booster route determined the anatomical site of the GC responses. Homologous subcutaneous (s.c.)/s.c. immunization increased GC induction in spleen and s.c. draining LNs, the ILNs, whereas s.c./intranasal (i.n.) immunization primarily induced GCs in mucosal draining LNs, the CLNs. The memory B cell composition was also route-dependent, with s.c./i.n. immunization preferentially generating GC-derived IgM^+^ memory B cells across lymphoid tissues. In contrast, homologous s.c./s.c. immunization promoted PC differentiation in spleen, yielding more BCMA^+^ cells especially in ILNs and BM, associated with elevated vaccine-specific serum IgG (days 7-14) and increased IgG ASCs in spleen and bone marrow. Heterologous s.c./i.n. immunization instead favored PC differentiation only in the CLNs and elevated serum and salivary IgA Abs. Correspondingly, BAFF-R and TACI expression was elevated on splenic GC B cells following s.c./s.c. immunization, whereas a higher expression was observed on CLNs GC B cells and IgM^+^ GC-derived memory cells after s.c./i.n. immunization. Together, these findings demonstrate that the booster immunization route directs the anatomical site of GC activity and determines the dominant GC-derived memory B cell subset. Homologous s.c./s.c. immunization maximizes systemic IgG responses, through enhanced BCMA-associated PC survival, whereas s.c./i.n. immunization promotes IgM^+^ GC-derived memory B cell induction while confining PC differentiation and BCMA expression to CLNs, resulting in combined systemic and mucosal Ab responses. These results support rational, route-informed early-life vaccine design to selectively enhance systemic IgG or combined systemic–mucosal antibody responses.

## 1 Introduction

Vaccines have significantly improved public health, preventing 146 million child deaths, including 101 million among infants (1). Despite these remarkable achievements, infectious diseases remain a leading cause of mortality in children under 5 years of age, highlighting the need for new vaccines, as well as enhanced efficacy of the existing vaccines through adjuvants or optimized delivery strategies. The neonatal immune system is poorly developed, making newborns especially susceptible to infections and less responsive to vaccines. In this age group, weak and short-lived antibody (Ab) responses to infection or vaccination are linked to limited germinal center (GC) activity and output, along with reduced plasma cell (PC) survival (2). T follicular helper (T_FH_) cells are a special subset of CD4 T cells that provide critical help to B cells within the GC reaction (3). Interactions of T_FH_ and B cells is essential for the formation and maintenance of GCs and for the generation of memory B cells (MBCs) and high-affinity Abs produced by PCs (4). Another CD4 T-cell subset involving GC regulation is the T follicular regulatory cell (T_FR_), which modulates humoral immune responses and promotes the generation of high-affinity, long-lived PCs (5). In contrast, MBC differentiation has been associated with weaker or more limited T_FH_-derived selection signals and may include lower-affinity clones, whereas differentiation into PCs is generally linked to stronger T cell help (6, 7). PCs that sustain long-lasting humoral immunity arise when plasmablasts exit the GCs, migrate to the bone marrow (BM), and successfully occupy specialized survival niches, where they mature into long-lived PCs. However, murine studies of early-life plasmablasts demonstrated that the neonatal BM microenvironment is less capable of supporting the establishment and persistence of long-lived PCs (8). Improving vaccination strategies using adjuvants and different immunization routes for priming and boosting has shown to affect both humoral and cellular immune responses (9–15). Adjuvants can enhance, modulate, and prolong immune responses (16, 17). In this study, we used a pneumococcal conjugate vaccine Pn1-CRM_197_ and two adjuvants CAF^®^01 and mmCT, priming 7 days old neonatal mice and administering a booster at 23 days of age (infant mice), as a model of early-life immunity that recapitulates key features of human neonatal and infant immune responses, as demonstrated previously (2, 10, 18–20). CAF01 is composed of a liposomal delivery vehicle constructed by the cationic surfactant dimethyldioctadecylammonium (DDA) containing the immunostimulator trehalose 6,6′-dibehenate (TDB). CAF01 signals via the C-type lectin receptor (CLR) Mincle and activates the Syk/Card9 pathway and production of pro-inflammatory cytokines (21–23). In adult humans and mice, CAF01 induces strong Th1/Th17 responses (11, 24). In early life, its combination with influenza hemagglutinin (HA) or RSV pre-fusion F protein vaccines has been shown to improve GC responses (25, 26). The adjuvant mmCT has also been shown to induce Th17 (27) and improve neonatal immune responses when given subcutaneously (s.c.) with pneumococcal conjugate vaccine (Pnc1-TT) or tetanus toxoid (TT) (28, 29). Additionally, when administered to neonatal mice intranasally (i.n.) with Pn1-CRM_197_, mmCT enhanced GC activation, vaccine-specific serum IgG and IgA, and salivary IgA and vaccine-specific IgG and IgA ASCs in spleen and BM, resulting in both enhanced induction and persistence of the immune response (13). Receptors belonging to the tumor necrosis factor receptor superfamily, including the B cell activating factor receptor (BAFF-R), transmembrane activator and cyclophilin ligand interactor (TACI), and B cell maturation antigen (BCMA), together with their ligands BAFF (B cell-activating factor) and APRIL (a proliferation-inducing ligand), are essential promoters of B cell survival and activation, thereby supporting humoral immunity. Engagement of BAFF-R by BAFF is critical not only for the development of follicular and marginal zone B cells and their persistence (30, 31) but also for GC formation, B cell selection, maintenance, and reactivation of memory B cells (32, 33). Furthermore, BAFF-R and TACI signaling pathways can promote immunoglobulin class-switch recombination (34, 35). BCMA interacts with both BAFF and APRIL but displays higher binding affinity for APRIL, and these interactions are particularly important for sustained survival of long-lived PCs (33, 36). Consistent with the importance of these receptors, neonates exhibit a lower proportion of newly formed B cells expressing BAFF-R, whereas higher proportions of B220^+^, marginal zone, and follicular B cells are BAFF-R^+^ compared with adults (37). This likely reflects the critical role of BAFF-R signaling in promoting the survival, maturation, and differentiation of newly formed B cells into marginal zone or follicular B cells, consistent with the higher proportion of newly formed relative to mature B cells in neonates (37). The TACI expression is also markedly lower in neonates than in adults, which likely contributes to their reduced antibody generation (38, 39). Notably, early-life immunization with Pnc1-TT and LT-K63 significantly increased the BAFF-R expression on these B cell subsets compared with both saline and vaccine-only control groups (37). Furthermore, our group previously demonstrated that neonates exhibit lower CD138 and BCMA expression on pre-plasmablasts/plasmablasts, which may contribute to their impaired differentiation into mature plasmablast/PC. Interestingly, the cells that do differentiate into mature plasmablast/PCs appear to be selectively enriched for BCMA expression, as BCMA levels within the plasmablast/PC compartment are proportionally higher than in adults (37).

Our previous work demonstrated distinct humoral responses induced by heterologous versus homologous prime-boost immunizations in early life up to 14 days and 35 days post-booster. Heterologous s.c./i.n. immunization with Pn1-CRM_197_ and CAF01 in s.c. priming and i.n. booster with mmCT adjuvant enhanced vaccine-specific IgG responses in systemic humoral compartments compared with i.n./i.n. immunization, while preserving mucosal IgA induction. In contrast, homologous s.c./s.c. immunization generated the strongest systemic IgG response overall, highlighting the importance of optimizing immunization routes to improve vaccine efficacy against respiratory pathogens (40). To further understand the mechanisms underlying these differences, we investigated whether heterologous and homologous immunization schedules had different effects on the induction of GC reactions and their output of MBCs and PCs at day 14 post-booster as in our previous study (40).

We show that prime-boost immunization routes strongly influence both the anatomical sites of GC induction and composition and distribution of GC-derived B cell populations. Homologous immunization promoted stronger PC induction, resulting in higher numbers of vaccine-specific IgG ASCs in spleen and BM along with higher serum Ab levels. In contrast, heterologous immunization primarily induced higher frequencies of IgM^+^ memory B cells across all tissues assessed.

## 2 Materials and methods

### 2.1 Mice

NMRI mice (5–6 weeks old) obtained from Taconic (Skensved, Denmark) were bred and housed in microisolator cages at the ArcticLAS vivarium facility in Reykjavík, Iceland. Animals were maintained under controlled conditions of temperature, light, and humidity, with free access to standard food and water. Breeding cages were checked daily, and pups remained with their mothers until weaning at 4 weeks of age. All procedures were approved by the Icelandic Experimental Animal Committee in accordance with regulation 279/2002.

### 2.2 Antigen, adjuvants, and immunization

The pneumococcal conjugate vaccine Pn1-CRM_197_, provided by Serum Institute of India (Pune, India), consists of a pneumococcal polysaccharide of serotype 1 (Pn1) conjugated to the genetically detoxified mutant protein of diphtheria toxin (DT) (CRM_197_) as a carrier (41). The ratio of polysaccharide (Pn1) to carrier (CRM_197_) was 1.1. The adjuvant mmCT, a non-toxic mutant of cholera toxin (CT), was produced as described (42) and provided by the Vaccine Research Institute (Gothenburg, Sweden). CAF01 (250 µg dimethyldioctadecylammonium and 50 µg trehalose dibehenate) was produced as previously described (43), consisting of glycolipid trehalose 6,6'-dibehenate (TDB) in cationic liposomes of the quaternary ammonium compound dimethyldioctadecylammonium (DDA) and provided by Statens Serum Institute (Copenhagen, Denmark).

The vaccine solutions were prepared 1 h before immunization. For s.c. priming 50 µL of vaccine solution (neonatal 7-day-old mice) and for s.c. booster immunization 100 µL of vaccine (infant 23-day-old mice) were injected at both sides of the base of tail. For i.n. immunization, 2 × 2.5 µL of vaccine solution for priming (neonatal 7-day-old mice) and 2 × 3 µL for booster (infant 23-day-old mice) were slowly delivered into the nares, with 30 min between doses. The amount of antigen used for immunization was 0.25 µg Pn1-CRM_197_ for s.c. priming, whereas heterologous groups received boosters of 0.25, 2, or 4 µg Pn1-CRM_197_ via the i.n. route and homologous groups received an s.c. booster of 0.25 µg Pn1-CRM_197_. Anesthesia was administered before i.n. booster but not before priming neonatal mice. Mice were euthanized 14 days after booster.

### 2.3 Blood and saliva sampling

Mice were bled from the tail vein 2 weeks after priming and weekly following the booster to measure Pn1-specific IgG and IgA levels in serum. Saliva was collected using absorbent sticks inserted into the mouth for 5 min and then transferred to PBS with a 10.0-µg/mL protease inhibitor (aprotinin; Sigma-Aldrich, St Louis, MO, USA) to prevent proteolysis. Saliva samples were pooled per group and stored at −70 °C.

### 2.4 Immunofluorescence staining and cell phenotyping by flow cytometry

Staining and flow cytometry were conducted after isolation of mononuclear cells 14 days post-booster, as previously described (44). A total of 20 µL of a 10^8^ cell stock (containing 10^6^ cells) was mixed with 490 µL of washing buffer (PBS with 0.5% BSA and 4 mM EDTA). The mixture was centrifuged at 300g for 5 min, and the supernatant was discarded. Fluorescently labeled antibodies against extracellular markers of interest were prepared in 50 µL of washing buffer containing Fc block (BD Biosciences), rat serum, and mouse serum (3% each) to minimize unspecific binding and added to the cells and incubated on ice for 30 min. Following incubation, cells were washed with 500 µL washing buffer, centrifuged again at 300g for 5 min, and resuspended in 100 µL washing buffer after removal of the supernatant. For intracellular staining, PBS was used in place of washing buffer in the preceding step; cells were fixed and permeabilized using the Cyto-Fast™ Fix/Perm Buffer Set (BioLegend) before staining with antibodies against intracellular markers of interest and finally resuspended in 100 µL washing buffer. The stained cells were analyzed using a BD FACSymphony™ A3 flow cytometer (BD Biosciences, San Jose, CA, USA), where 1,000,000 events were recorded for spleen, BM, and ILN samples whereas 500,000 events were recorded for CLN samples. Data analysis was done using Kaluza^®^ software (version 2.2 from Beckman Coulter, Beckman Coulter). Dead cells and doublets were excluded. Our antibody panel included mBCMA FITC (clone 161616; R&D Systems). From BD Biosciences, we used TACI/CD267 AlexaFluor 647 (8F10), CD80/B7–1 R718 (16-10A1), (C10-1), CD45R/B220 BV480 (RA3-6132), CD73 BV711 (TY-11.8), IgM BV786 (R6-60.2), IgD(b) BUV496 (217-170), CD38 BUV395 (90/CD38), CD268/BAFF-R (7H22-E16), GL7 “T and B cell activation antigen” PE (GL7), and CD95 PE-CY7 (Jo2). From BioLegend, we included CD138/syndecan-1 PE-Dazzle 594 (281-2). Gating strategies and the total number of cell populations assessed are included in the **Supplementary Material** (**Supplementary Figures 1–5**).

### 2.5 Enzyme-linked immunosorbent assay (ELISA)

To assess specific humoral responses to the polysaccharide (Pn1) part of the conjugate vaccine Pn1-CRM_197_, anti-Pn1 IgG and IgA in serum and saliva were measured by ELISA as described previously (10). Briefly, microtiter plates (MaxiSorp; Nunc AS, Roskilde, Denmark) were coated with 5 µg/mL of Pn1 (American Type Culture Collection, Rockville, MD) in PBS for 5 h at 37 °C, the plates were washed and blocked with PBS with 0.05% Tween 20 (Sigma) containing 1% BSA (Sigma). Serum samples and standard were neutralized with cell wall polysaccharide (CWPS; Statens Serum Institute), diluted 1:50 (1:25 for saliva) in PBS with 0.05% Tween 20, and incubated in 500 mg/mL of CWPS for 30 min at room temperature. Neutralized serum samples and standard were serially diluted, whereas saliva samples were measured undiluted; both were incubated for 2 h in duplicates in 100 µL/well in Pn1-coated microtiter plates at room temperature. The plates were washed and then incubated with horseradish peroxidase-conjugated goat anti-mouse IgG or IgA (Southern Biotechnology Associates, Birmingham, AL) diluted 1:5,000 in PBS-Tween 20 for 2 h at room temperature in 100 µL/well. The plates were washed, and 3,3′,5,5′-tetramethylbenzidine-substrate (Kirkegaard & Perry Laboratories, Gaithersburg, MD) was used for development and the reaction stopped with 0.18 M H_2_SO_4_. Absorbance was measured at an optical density of 450 nm in Multiskan FC Microplate Photometer (Thermo Scientific, Waltham, Massachusetts, USA). The standard for Pn1-specific Abs was prepared by pooling sera from adult mice hyperimmunized with the conjugate vaccine. The results were calculated from a standard curve and expressed as mean log of ELISA units (EU)/mL for IgG and mean of EU/mL for IgA.

### 2.6 Enzyme-linked immunospot, ELISpot

Pn1-specific IgG and IgA ASCs in spleen and BM were enumerated by ELISpot, 14 days post-booster after isolation of mononuclear cells, as previously described (45). MultiScreen high protein binding immobilon-P membrane plates (Millipore Corporation, Bedford, MA) were coated with 10 µg/mL Pn1 overnight at 37 °C in 50 µL/well, blocked with complete RPMI 1640 (Life Technologies BRL, Life Technologies, Paisley, U.K.) containing 25 mM HEPES buffer (Gibco™), 2 mM L-glutamine (Gibco™), 100 U/mL penicillin/100 µg/mL streptomycin (Gibco™), and 10% fetal calf serum (FCS; Gibco™). Duplicates of cells from spleen and BM were incubated in four threefold dilutions starting with 1 × 10^7^ cells in 100 µL of complete RPMI 1640 per well for 5 h at 37 °C, washed and incubated with alkaline-phosphatase (ALP) conjugated goat anti-mouse IgG or IgA (Southern Biotechnology Associates) overnight at 4 °C, and developed by 5-bromo-4-chloro-3-indolylphosphate and NBT in AP development buffer (Bio-Rad Labs, Hercules, CA). Numbers of spots (each representing a cell secreting specific Abs) were counted by ELISPOT reader ImmunoSpot R S6 Ultimate using ImmunoSpot R Software (Cellular Technology Limited (CTL) Europe, Bonn, Germany).

### 2.7 Statistical analysis

Statistical analyses between immunization groups at each time point were first assessed using the Kruskal–Wallis test, and where significant differences were found, comparisons were performed using the Mann–Whitney U test between two groups (*p ≤ 0.05, **p ≤ 0.01, ***p ≤ 0.001), with p-values < 0.05 considered statistically significant. All analyses were conducted using GraphPad Prism 9.0 (GraphPad Software, La Jolla, CA).

## 3 Results

### 3.1 The booster route strongly determines the site of germinal center induction

**Figure 1:**
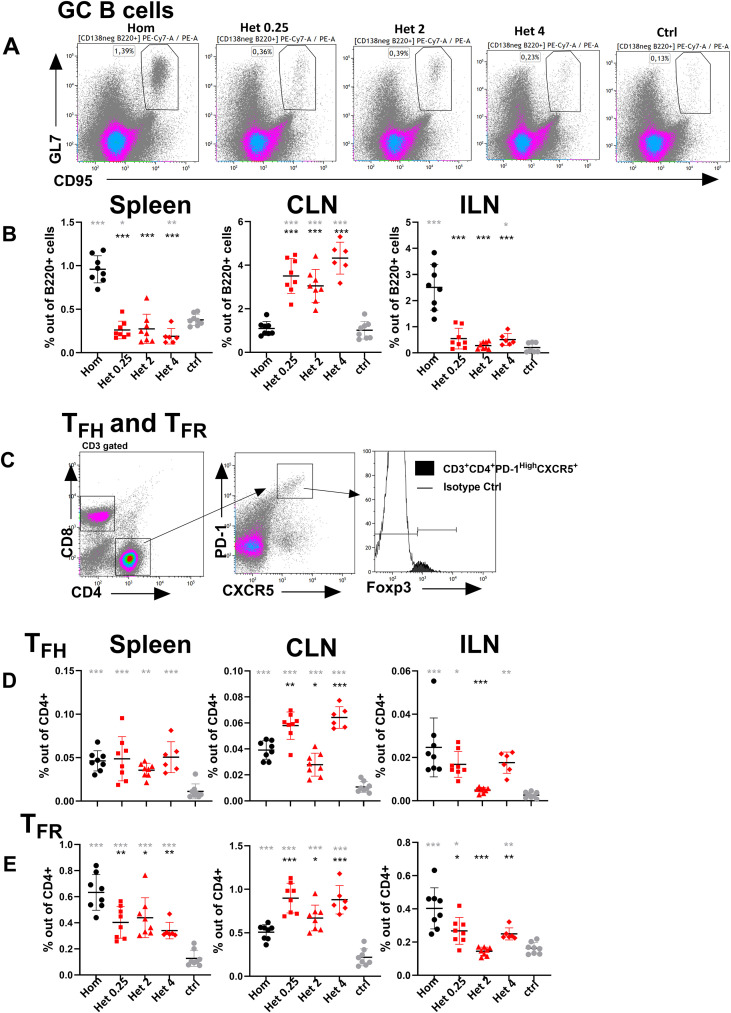
The route of booster immunization strongly determines the site of germinal center induction. **(A)** Dot plot representative of GC B cells in spleen. **(B)** GC B cell frequencies in spleen, CLNs, and ILNs 14 days post-booster. **(C)** Dot plot representative of T_FH_ and T_FR_ frequencies in spleen 14 days post-booster. **(D)** T_FH_ frequencies in spleen, CLNs, and ILNs 14 days post-booster. **(E)** T_FR_ frequencies in spleen, CLNs, and ILNs 14 days post-booster. Mice were immunized by different immunization schedules utilizing 0.25, 2, or 4 µg of Pn1-CRM197, CAF01, and 2 µg of mmCT. Each symbol represents one mouse, and results are shown as means ± SD in 6–8 mice per group. For statistical evaluation, the Kruskal–Wallis test was first applied and then Mann–Whitney U-test was used to compare each two groups at a time. Black stars represent p values after comparison of homologous s.c./s.c. group to heterologous s.c./i.n. groups, and gray stars represent comparisons of all the groups to the control group. *p ≤ 0.05, **p ≤ 0.01, ***p ≤ 0.001. Hom (Homologous s.c./s.c.) black circles, Het 0.25 (heterologous s.c./i.n. 0.25 µg of Pn1-CRM_197_) red boxes, Het 2 (heterologous s.c./i.n. 2 µg of Pn1-CRM_197_) red triangles, Het 4 (heterologous s.c./i.n. 4 µg of Pn1-CRM_197_) red rhombus, and ctrl (control) gray circles.

To evaluate how the booster route in heterologous versus homologous immunization schedule impacts the GC reaction, one of the key limiting factors in early-life humoral responses, GC B cells and other critical GC-associated cell populations, including T_FH_ and T_FR_ cells, were assessed 14 days after booster immunization in spleen and draining lymph nodes (CLNs for the mucosal booster and ILNs for the s.c. booster) (**Figure 1**; **Supplementary Figure 2**). Mice were immunized with Pn1-CRM_197_ using either a heterologous schedule consisting of s.c. priming (7 day old mice) followed by i.n. booster 16 days after priming (23 days old mice) or a homologous schedule with s.c. priming and s.c. booster. We also evaluated whether increasing the Pn1-CRM_197_ dose in the i.n. booster affected GC induction and the fate of responding B cells. This was based on our previous observations that limited systemic primary and booster Ab and ASC responses to i.n. immunization can be enhanced by increasing the Pn1-CRM_197_ dose (13, 40), likely due to enhanced antigen uptake in the respiratory mucosa. Thus, all groups received 0.25 µg Pn1-CRM_197_ with CAF01 adjuvant for s.c. priming, whereas heterologous groups received boosters of 0.25, 2, or 4 µg Pn1-CRM_197_ with mmCT adjuvant via the i.n. route, whereas homologous groups received a 0.25-µg Pn1-CRM_197_ booster with mmCT administered s.c. Age-matched unimmunized mice were used as controls.

The booster route determined the location of the GC induction, where the homologous s.c./s.c. immunization promoted GC formation in the subcutaneous draining lymph nodes (ILNs) but also systemically in the spleen as the frequency of GC B cells was higher in spleen following homologous than any of the heterologous immunizations. Conversely, all heterologous s.c./i.n. immunization schedules induced GC B cells exclusively in the mucosal draining lymph nodes (CLNs) (**Figures 1A, B**). We observed that T_FR_ induction correlated with the sites of GC formation, which varied depending on the booster route, whereas T_FH_ differentiation showed a less clear relationship as their differentiation only correlated with GC B cells in ILN and CLN but not in spleen (**Supplementary Figure 6**). T_FR_ frequencies were higher in spleen and ILNs following homologous compared with a heterologous immunization schedule, whereas in the CLNs their frequency was increased after i.n. boosters (**Figures 1C–E**).

These results highlight that the booster route not only modulates the magnitude of GC induction but also determines its anatomical localization. Homologous immunization preferentially drives systemic responses in spleen and the s.c. draining lymph nodes (ILNs), whereas heterologous immunization favors the mucosal-draining lymph node (CLNs). This compartmentalization provides insight into how prime-boost immunization strategies can be tailored to direct humoral immunity toward systemic or mucosal protection.

### 3.2 Heterologous prime-boost immunization preferentially promotes the induction of unswitched IgM^+^ memory B cells in all lymphoid organs

**Figure 2:**
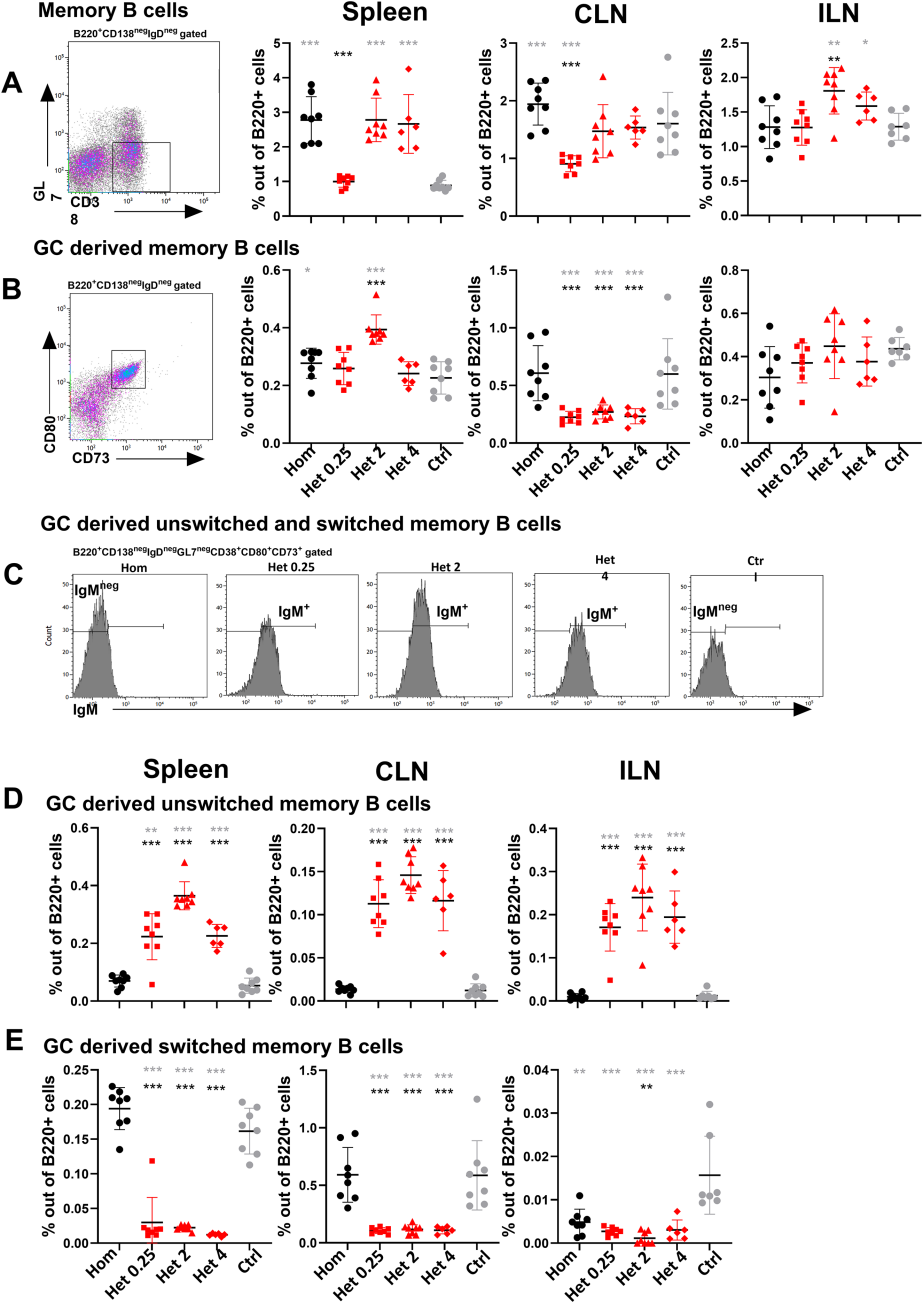
Heterologous prime-boost strategy preferentially promotes the induction of unswitched IgM^+^ memory B cells in all lymphoid organs. **(A)** Dot plot representative of memory B cells in spleen and frequencies of memory B cells in spleen, CLNs, and ILN 14 days post-booster. **(B)** Dot plot representative of GC-derived memory B cells in spleen and frequencies of GC-derived memory B cells in spleen, CLNs, and ILNs 14 days post-booster. **(C)** Dot plot representative of GC-derived unswitched and switched memory B cells in spleen 14 days post-booster. **(D)** Frequencies of GC-derived unswitched memory B cells in spleen, CLNs, and ILNs 14 days post-booster. **(E)** Frequencies of GC-derived switched memory B cells in spleen, CLNs, and ILNs 14 days post-booster. Mice were immunized by different immunization schedules utilizing 0.25, 2, or 4 µg of Pn1-CRM197, CAF01, and 2 µg of mmCT. Each symbol represents one mouse, and results are shown as means ± SD in six to eight mice per group. For statistical evaluation, the Kruskal–Wallis test was first applied and then Mann–Whitney U-test was used to compare each two groups at a time. Black stars represent p values after comparison of homologous s.c./s.c. group to heterologous s.c./i.n. groups, and gray stars represent comparisons of all the groups to the control group. *p ≤ 0.05, **p ≤ 0.01, ***p ≤ 0.001. Hom (homologous s.c./s.c.) black circles, Het 0.25 (heterologous s.c./i.n. 0.25 µg of Pn1-CRM_197_) red boxes, Het 2 (heterologous s.c./i.n. 2 µg of Pn1-CRM_197_) red triangles, Het 4 (heterologous s.c./i.n. 4 µg of Pn1-CRM_197_) red rhombus, and ctrl (control) gray circles.

Given the compartmentalized GC responses described above, we next characterized the effect of the different immunization regimens on GC output of MBCs in spleen, CLN, and ILN 14 days after booster immunization. Specifically, we compared the frequencies of MBCs and GC-derived MBCs across different immunization schedules and determined whether these populations were unswitched (IgM^+^) or class-switched (IgM^neg^) (**Figure 2**; **Supplementary Figure 3**).

We observed that both homologous s.c./s.c. and heterologous s.c./i.n. schedules induced comparable overall frequencies of memory B cells in spleen, CLNs, and ILNs (**Figure 2A**). An exception was observed at the lowest antigen dose assessed (0.25 µg Pn1-CRM_197_), where the i.n. booster resulted in a significantly lower frequency of MBCs in CLNs and spleen compared with the s.c. booster with the same dose (**Figure 2A**). This indicates that higher antigen doses are required for i.n. immunization than s.c. immunization to achieve comparable MBC induction, except in the s.c. draining LNs (ILNs). These findings are consistent with previous reports demonstrating that higher i.n. doses of Pn1-CRM_197_ are required to elicit robust ASC and Ab responses (13, 40). Although heterologous immunization did not clearly enhance GC-derived memory B cells (**Figure 2B**), interestingly, analysis of GC-derived memory B cell subpopulations revealed higher frequencies of GC-derived unswitched IgM^+^ MBCs following all heterologous immunizations compared with homologous s.c./s.c. immunization in all lymphoid tissue assessed, spleen, CLNs, and ILNs (**Figures 2C–E**). Furthermore, in the CLNs, GC-derived unswitched MBCs positively correlated with both T_FH_ and T_FR_ frequencies (**Supplementary Figure 7**).

Taken together, heterologous s.c./i.n. immunization favors the generation of unswitched IgM^+^ MBCs across all tissues assessed, irrespective of vaccine dose. These findings underscore that a mucosal booster after s.c. priming preserves the unswitched MBC pool that potentially maintains broader specificity. Whether this represents an advantage or disadvantage remains to be further explored.

### 3.3 Homologous immunization schedule promotes plasma cell differentiation that parallels with elevated vaccine-specific IgG responses

**Figure 3:**
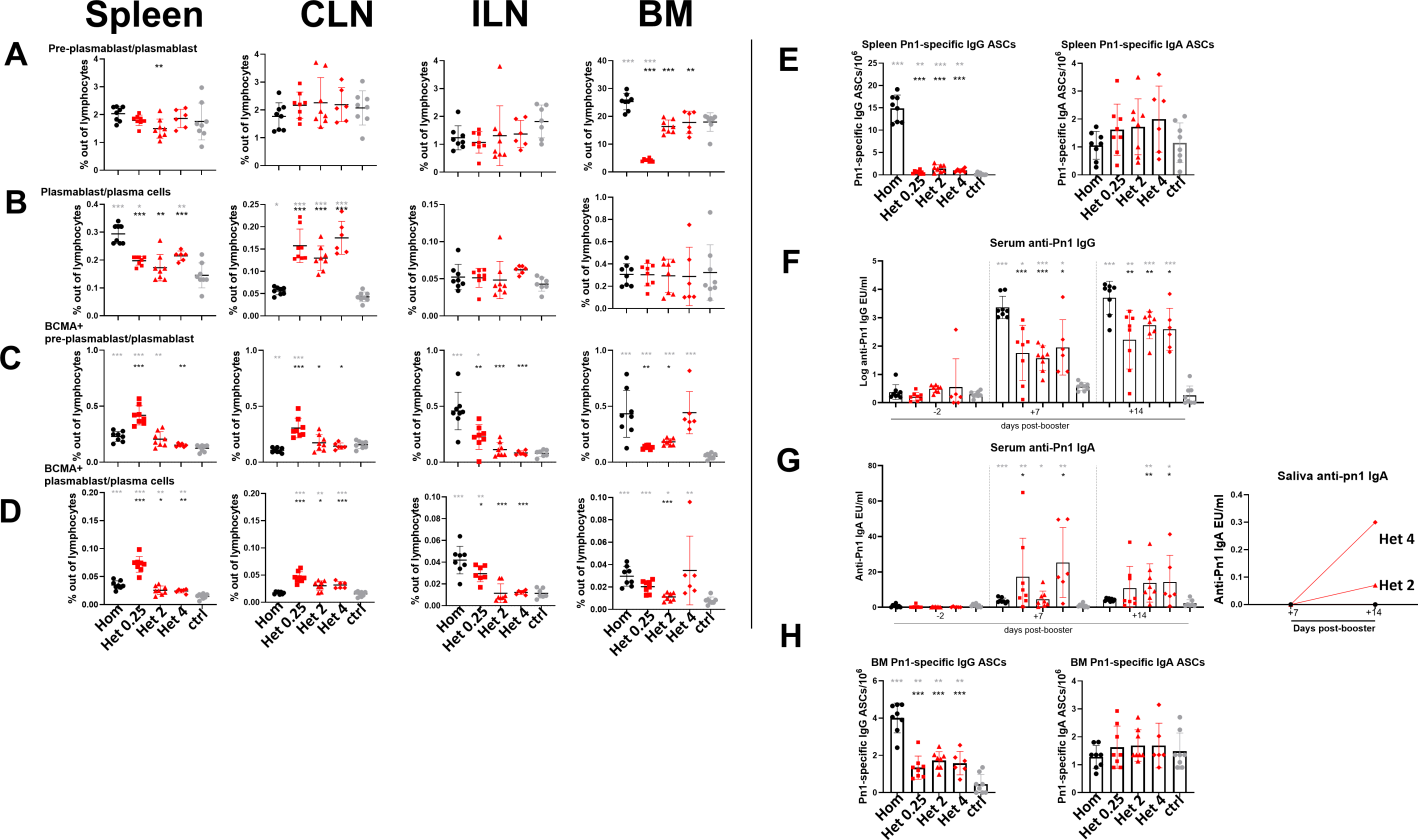
Homologous immunization promotes plasma cell differentiation and associates with elevated vaccine-specific IgG responses. **(A)** Frequencies of pre-plasmablast/plasmablast (CD138^int^B220^+^), plasmablast/plasma cells (CD138^hi^B220^−/+^) in spleen 14 days post-booster. **(B)** Frequencies of pre-plasmablast/plasmablast and plasmablast/plasma cells in CLNs 14 days post-booster. **(C)** Frequencies of pre-plasmablast/plasmablast, plasmablast/plasma cells, and BCMA expression in ILNs 14 days post-booster. **(D)** Frequencies of pre-plasmablast/plasmablast, plasmablast/plasma cells, and BCMA expression in BM 14 days post-booster. **(E, H)** Pn1-specific IgG+ or IgA ASCs in spleen and BM 14 days post-booster. **(F, G)** Serum anti-Pn1 IgG or IgA levels at −2 to 14 days post-booster in serum and saliva. Mice were immunized by different immunization schedules utilizing 0.25, 2, or 4 µg of Pn1-CRM197, CAF01, and 2 µg of mmCT. Each symbol represents one mouse, and results are shown as means ± SD in six to eight mice per group. For statistical evaluation, the Kruskal–Wallis test was first applied and then Mann–Whitney U-test was used to compare each two groups at a time. Black stars represent p values after comparison of homologous s.c./s.c. group to heterologous s.c./i.n. groups, and gray stars represent comparisons of all the groups to the control group. *p ≤ 0.05, **p ≤ 0.01, ***p ≤ 0.001. Hom (homologous s.c./s.c.) black circles, Het 0.25 (heterologous s.c./i.n. 0.25 µg of Pn1-CRM_197_) red boxes, Het 2 (heterologous s.c./i.n. 2 µg of Pn1-CRM_197_) red triangles, Het 4 (heterologous s.c./i.n. 4 µg of Pn1-CRM_197_) red rhombus, and ctrl (control) gray circles.

To determine whether the differences in GC and MBC composition described above also affected differentiation toward ASCs as observed in a previous study (40), we analyzed plasmablast and PC populations and their BCMA expression in spleen, CLN, ILN, and BM 14 days after booster immunization (**Figures 3A–D**; **Supplementary Figure 4**). Vaccine-specific IgG and IgA ASCs in spleen and BM were also assessed (**Figures 3E–H**). Two populations were defined, pre-plasmablast/plasmablast as B220^+^CD138^int^ and plasmablast/PC as B220^+/−^CD138^high^, like in our previous study, where we observed a decreased ratio between these populations compared with adults, along with a lower expression of CD138 (34).

The frequency of plasmablast/PC in spleen was higher following homologous than heterologous immunization (**Figure 3B**), suggesting that homologous immunization promotes more effectively the B cell differentiation into fully developed antibody-secreting cells as previously reported (40). This associated with higher frequencies of BCMA^+^ pre-plasmablast/plasmablast and plasmablasts/PC in ILNs (**Figures 3C, D**), and in BM for total number following homologous s.c./s.c. immunization with the exception of the heterologous group receiving 4 µg Pn1-CRM_197_ booster (**Supplementary Figure 4**). In contrast, PC frequencies and their expression of BCMA in CLNs were higher after heterologous s.c./i.n. than homologous s.c./s.c. immunization, indicating that i.n. delivery preferentially drives local ASC differentiation in the mucosal-draining sites (**Figures 3B** CLNs). These phenotypic differences were reflected functionally, as homologous s.c./s.c. immunization induced significantly higher titers of Pn1-specific serum IgG titers from day 7 to day 14 post-booster (**Figure 3F**) and higher number of vaccine-specific IgG ASCs in both spleen and BM (**Figures 3E, H**). In contrast, heterologous s.c./i.n. immunization induced higher serum IgA titers, particularly with the 2 and 4 µg Pn1-CRM_197_ boosters and uniquely elicited salivary IgA responses at day 14 post-booster (**Figure 3G**).

Together with the GC and MBC data, these findings indicate that the booster route directs not only the magnitude and anatomical location of the GC induction and composition of the MBC output but also the differentiation and isotype profile of ASCs. Homologous s.c./s.c. immunization favors systemic immunity, driving robust IgG ASC generation in spleen and BM with higher BCMA^+^ PCs in ILNs and slightly in BM possibly associated with enhanced survival, as observed in our previous study 35 days post-booster (40). In contrast, heterologous s.c./i.n. immunization favors the generation of unswitched MBCs and directs ASC differentiation primarily in the mucosal draining CLN, thereby promoting more balanced IgG and IgA responses, including mucosal IgA secretion in saliva.

### 3.4 BAFF-R/TACI upregulation on germinal center and memory B cells aligns with a booster administration route

**Figure 4:**
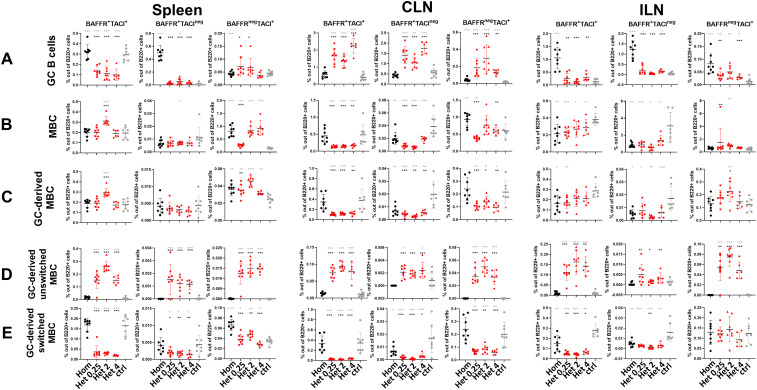
The route of booster determines BAFF-R/TACI upregulation. **(A)** Frequency of BAFFR and TACI expression on GC B cells in spleen, CLNs and ILNs 14 days post-booster. **(B)** Frequency of BAFFR and TACI expression on memory B cells in spleen, CLNs and ILNs 14 days post-booster. **(C)** Frequency of BAFFR and TACI expression on GC-derived memory B cells in spleen, CLNs and ILNs 14 days post-booster. **(D)** Frequency of BAFFR and TACI expression on GC-derived unswitched memory B cells in spleen, CLNs and ILNs 14 days post-booster. **(E)** Frequency of BAFFR and TACI expression on GC-derived switched memory B cells in spleen, CLNs and ILNs 14 days post-booster. Mice were immunized by different immunization schedules utilizing 0.25, 2 or 4 µg of Pn1-CRM197, CAF01, and 2 µg of mmCT. Each symbol represents one mouse and results are shown as means ± SD in 6-8 mice per group. For statistical evaluation, the Kruskal–Wallis test was first applied and then Mann–Whitney U-test was used to compare each two groups at a time. Black stars represent p values after comparison of homologous s.c./s.c. group to heterologous s.c./i.n. groups and grey stars represent comparisons of all the groups to the control group. *p ≤ 0.05, **p ≤ 0.01, ***p ≤ 0.001. Hom (Homologous s.c./s.c.) black circles, Het 0.25 (Heterologus s.c./ i.n. 0.25 µg of Pn1-CRM_197_) red boxes, Het 2 (Heterologus s.c./ i.n. 2 µg of Pn1-CRM_197_) red triangles, Het 4 (Heterologus s.c./ i.n. 4 µg of Pn1-CRM_197_) red rhombus and ctrl (control) grey circles.

BAFF-R delivers BAFF-dependent survival and selection signals that sustain GC B cells and help maintain the MBC pool (30, 31). In contrast, when engaged by BAFF or APRIL, TACI promotes immunoglobulin class-switch recombination, particularly to IgG and IgA via MyD88, and regulates B cell homeostasis (34, 35). To investigate whether the immunization regimens influenced these pathways, we profiled BAFF-R and TACI expression on GC B cells and MBCs (**Figure 4**; **Supplementary Figure 5**).

BAFF-R and TACI expressions were higher on GC B cells following homologous than heterologous immunization both systemically in the spleen and in the s.c. draining lymph nodes (ILNs). In contrast, after the i.n. booster in heterologous schedules, a higher expression was observed in the mucosal draining lymph nodes (CLNs) only (**Figure 4A**). Interestingly, BAFF-R and TACI expression on unswitched GC-derived MBCs was higher after heterologous than homologous immunization across all lymphoid tissues assessed, including spleen, CLNs, and ILNs (**Figure 4D**).

Overall, the booster route aligned with upregulation of BAFF-R and TACI expression across GC B cells and GC-derived MBC subsets. Homologous s.c./s.c. immunization resulted in higher BAFF-R/TACI expression on GC B cells predominantly in spleen and ILNs, whereas heterologous s.c./i.n. immunization upregulation was observed on GC B cells in the CLNs. In addition, heterologous boosting was associated with increased BAFF-R/TACI expression on IgM^+^ GC-derived MBCs across the lymphoid tissues analyzed. These findings indicate a route-dependent distribution of BAFF-family receptor expression.

## 4 Discussion

In this study, we demonstrate that the immunization strategy, and particularly the route of booster delivery, profoundly shapes multiple aspects of the B cell response. Homologous s.c./s.c. immunization was associated with stronger systemic GC induction and features of PC differentiation, whereas heterologous s.c./i.n. immunization redirected humoral responses toward mucosal draining lymph nodes and was accompanied by a higher output of GC-derived IgM^+^ MBCs across all lymphoid tissues at day 14 post-booster.

In our neonatal mouse model, homologous s.c./s.c. immunization induced stronger GC responses in the spleen and ILNs, whereas heterologous s.c./i.n. immunizations redirected GC induction to the mucosal draining lymph nodes (CLNs) (**Figure 1**). Consistent with this, we previously reported that the frequencies of both IgG and IgA ASCs were higher in CLNs following heterologous than homologous immunization 14 days after booster immunization (40). Together, these observations indicate that, under the heterologous s.c./i.n. regimen and adjuvants used, CLNs act as key inductive sites for the early generation of IgG and IgA responses (37). This interpretation is in line with earlier work demonstrating that surgical removal of CLNs but not nasal-associated lymphoid tissue (NALT) in 8 days old mice prior to i.n. immunization (2 days later) with a different pneumococcal conjugate vaccine plus IL-12 markedly impaired mucosal IgA production, systemic antibody responses, and protection against pneumococcal colonization following intranasal immunization (46). Collectively, these data support a central role for CLNs in orchestrating both mucosal IgA responses and systemic antibody production following mucosal booster. In our study, T_FH_ and particularly T_FR_ frequencies correlated with GC activity, with stronger correlations observed in the spleen and ILN following homologous s.c./s.c., whereas the strongest correlations were observed in CLNs following heterologous s.c./i.n. immunization (**Figure 1**; **Supplementary Figure 6**). In line with our findings, T_FR_ cells have been reported to modulate GC magnitude and quality to promote antigen-specific GC B cell responses during influenza virus infection (5). Following viral challenge, T_FR_ cells were required for strong generation of virus-specific, long-lived PCs; for Ab production against the major influenza virus glycoproteins, hemagglutinin (HA), and neuraminidase (NA); and for proper regulation of the BCR repertoire (5).

Importantly, we demonstrated that heterologous s.c./i.n. immunization preferentially generated GC-derived IgM^+^ unswitched MBCs (**Figure 2**). In this study, we did not directly assess recall responses, functional differentiation capacity, affinity maturation, avidity, or somatic hypermutation levels of IgM^+^ GC-derived memory B cells. Thus, our interpretation of their potential contribution to rapid effector responses and protective immunity is based on previously published studies demonstrating the functional importance of this memory B cell subset. Studies using a tetracycline-regulated pulse-chase system to track population turnover over 402 days suggested that IgM^+^ memory B cells are particularly poised to initiate GC induction during secondary responses (47). Similarly, in a study using PE-conjugated B cell tetramers to track *Plasmodium*-specific MBCs, somatically hypermutated IgM^+^ MBCs were the earliest responders upon secondary challenge, as they differentiated rapidly into T-cell–dependent B220^+^CD138^+^ plasmablasts (producing both IgM and IgG) as well as T-cell–independent B220^-^CD138^+^IgM^+^ PCs, even in the presence of switched MBCs (48). Together, these findings indicate that IgM^+^ memory B cells serve as a rapid, plastic effector source during early recall responses, whereas switched MBCs contribute more potently to sustained systemic responses. Clinical observations in humans further support the importance of IgM^+^ MBCs, as IgM^+^CD27^+^ MBCs play a critical role in protection against encapsulated bacteria and seem to be largely generated or maintained in the spleen (49). Consistent with this, splenectomized children and patients with congenital asplenia exhibit markedly reduced numbers of circulating IgM^+^ MBCs along with increased susceptibility to *Streptococcus pneumoniae*, supporting the idea that this subset of MBCs provides rapid, T-cell–independent recall responses to polysaccharide antigens or encapsulated bacteria (49). Beyond bacterial infections, deficiencies in IgM MBCs have been associated with severe outcomes in viral infections, including COVID-19, whereas having IgM MBC responses is associated with milder disease courses. Importantly, studies in patients with common variable immunodeficiency (CVID) or splenectomized patients demonstrate that IgM MBC deficiency correlates with the absence of intestinal IgA^+^ PCs, suggesting that IgM MBCs represent an important source of mucosal IgA production in addition to contributing to systemic responses (50). Supporting this notion, TLR9 and TACI signaling has been shown to drive differentiation of IgM MBCs into IgA^+^ PCs *in vitro* (51).

Importantly, IgM^+^ MBCs are functionally heterogeneous, with subsets defined by CD80 and PD-L2 expression exhibiting distinct functional states that are not strictly determined by isotype (49). This supports the concept that MBC populations can be biased toward different recall trajectories, including rapid effector differentiation or alternative recall programs (49) (49).Using an AID-dependent memory B cell labeling model, it has been demonstrated that immunization generates multiple MBC populations that persist for months, including IgM^+^ and IgG1^+^ subsets in GC-like structures and outside follicles. Upon secondary challenge, IgG1^+^ MBCs preferentially differentiated into plasma cells, whereas IgM^+^ MBCs reinitiated germinal center reactions, supporting a layered model of memory with distinct recall functions (52). In a *P. yoelii* 17X mouse model, it was shown by bulk phenotyping and antigen-specific tetramer enrichment to track long-lived MBC subsets over time, that the MBC compartment is highly heterogeneous and not defined by isotype alone. Major splenic MBC populations are defined by IgM status and CD73/CD80 co-expression. Upon adoptive transfer followed by infection, both IgM^+^ and IgM^−^ CD73^+^CD80^+^ MBCs generated plasmablasts secreting antigen-specific Abs and differentiated into GC-like cells, demonstrating dual recall potential within each isotype-defined memory pool. Antigen-specific rechallenge confirmed that plasmablast output was dominated by IgM^−^, whereas IgM^+^ MBC cells also contributed, emphasizing that recall fate is subset-dependent rather than simply “IgM vs. IgG”. Together, IgM^+^ MBCs represent a flexible reservoir capable of both rapid effector differentiation and secondary GC participation, depending on the recall context (53).

Collectively, our data indicate that heterologous s.c./i.n. immunization may preferentially expand IgM^+^ GC-derived MBCs, a population uniquely poised to provide rapid effector responses upon secondary antigen exposure. These cells retain the capacity to re-enter GCs and fuel secondary GC reactions or to home to mucosal sites where they can differentiate into IgA-secreting PCs. Through these mechanisms, IgM^+^ MBCs may contribute to both systemic and mucosal immunity. In contrast, homologous s.c./s.c. immunization favors the generation of sustained long-lived systemic Ab responses. Whether these distinct memory B cell programs ultimately confer an advantage or disadvantage to protective immunity remains to be determined and is beyond the scope of the present study.

During early life, most plasmablasts can home to the BM, but they often fail to differentiate into long-lived PCs due to limited survival signals, particularly APRIL, resulting in transient Ab responses (54). In previous studies, our group demonstrated that the adjuvants LT-K63 and mmCT, when administered with pneumococcal conjugate vaccine or tetanus toxoid (TT), upregulate BCMA expression on plasmablasts/PCs in both the spleen and BM (29, 37). These adjuvant and vaccine combinations also increased the secretion of APRIL in BM (29, 37), and elevated BCMA expression may support prolonged PC survival by facilitating APRIL binding (36). Consistent with these observations, we previously showed that homologous s.c./s.c. immunization induces stronger humoral systemic IgG responses than heterologous s.c./i.n. immunizations, whereas heterologous immunizations promotes more pronounced humoral IgA responses both mucosally and systemically (40). Plasmablast/PC frequencies were higher in spleen following homologous s.c./s.c., with a greater fraction of BCMA^+^ cells in ILNs and to some extent in the BM, features associated with PC survival. Functionally, homologous s.c./s.c. immunization elicited higher Pn1-specific serum IgG titers (days 7–14) and greater numbers of vaccine-specific IgG ASCs in spleen and BM. However, this was not reflected in higher frequency or total number of BCMA^+^ cells, although they tended to be higher than some of the heterologous s.c./i.n. groups (**Figure 3**; **Supplementary Figure 4**). In contrast, heterologous s.c./i.n. immunization preferentially increased the frequency of BCMA^+^ cells in CLN and resulted in higher numbers of vaccine-specific IgG and IgA ASCs in this organ, consistent with the serum and salivary IgA responses (**Figure 3**). BCMA signaling supports PC survival in both mice and humans, and recent genetic studies in mice confirm that BCMA and/or TACI are required for PC maintenance (36, 55). In addition, both TACI and BAFF-R can promote immunoglobulin class switching (34). BAFF-R deficiency in humans leads to markedly reduced circulating B cells and low IgM and IgG serum concentrations, whereas IgA levels remain normal or elevated (56). In our study, BAFF-R and TACI expression on GC B cells was higher in the spleen and ILNs following homologous s.c./s.c. immunization, whereas expression was increased in CLNs following heterologous s.c./i.n. immunization. Within the memory compartment, a larger fraction of IgM^+^ GC-derived MBCs expressed BAFF-R/TACI following s.c./i.n. regimen than after s.c./s.c. immunization (**Figure 4**). Importantly, our analysis measured BAFF-R and TACI expression but did not directly test BAFF/APRIL signaling. Therefore, the observed upregulation likely reflects differences in activation or survival states across tissues rather than direct evidence that BAFF-R/TACI signaling directly determines B cell fate. On that note, both IgM^+^ and IgG1^+^ memory B cells require BAFF and BAFFR for long-term survival, as their absence reduces memory B cell numbers and impairs recall Ab responses (32). Consistently, BAFFR regulates early memory B cell responses and activated B cells maintain BAFFR expression after differentiation into GC or memory B cells in mice, with BAFF/BAFFR signaling being essential for optimal memory B cell maintenance (57).

Overall, our findings demonstrate that following the same neonatal s.c. priming, altering the booster route substantially reshapes the humoral immune responses. Homologous s.c./s.c. immunization promotes systemic GC induction in spleen and ILNs, enriches for BCMA^+^ PCs in ILNs, and generates strong Pn1-specific serum IgG responses with increased IgG ASCs in spleen and BM. In contrast, heterologous s.c./i.n. boosting redirects GCs to CLNs, preferentially generates BAFF-R/TACI^+^ IgM^+^ GC-derived MBCs across lymphoid tissues, and promotes PC differentiation and IgA-dominated responses in the mucosal-draining lymph nodes, CLN, and saliva. Importantly, these data reveal that the booster route can simultaneously influence GC localization, memory B cell fate, and the balance between systemic and mucosal humoral immunity.

## Data Availability

The original contributions presented in the study are included in the article/**Supplementary Material**. Further inquiries can be directed to the corresponding author.
