# Supplementary material for: Parenteral neonatal priming followed by heterologous mucosal booster favors IgM+ memory B cell induction over systemic plasma cell differentiation
Source: Front Immunol. 2026 May 5;17:1792155. doi: 10.3389/fimmu.2026.1792155 (PMC13183861; doi:10.3389/fimmu.2026.1792155)
Supplement: Supplementary file 1 [file DataSheet1.pdf]

Supplementary Material

Parenteral neonatal priming followed by heterologous mucosal booster favors IgM^+ memory B cell induction over systemic plasma cell differentiation

Poorya Foroutan Pajoohian[1,2], Audur Anna Aradottir Pind[1,2], Jenny Lorena Molina Estupiñan[1,2], Dennis Christensen[3], Gabriel Kristian Pedersen[3], Thorunn A. Olafsdottir[1], Ingileif Jonsdottir[1], Stefania P. Bjarnarson[1,2*]

[1]Faculty of Medicine, Biomedical Center, School of Health Sciences, University of Iceland, Reykjavik, Iceland

[2]Department of Immunology, Landspitali, the National University Hospital of Iceland, Reykjavik, Iceland

[3] Center for Vaccine Research, Statens Serum Institut, Copenhagen, Denmark.

*** Correspondence:**
Stefania P. Bjarnarson
stefbja@landspitali.is

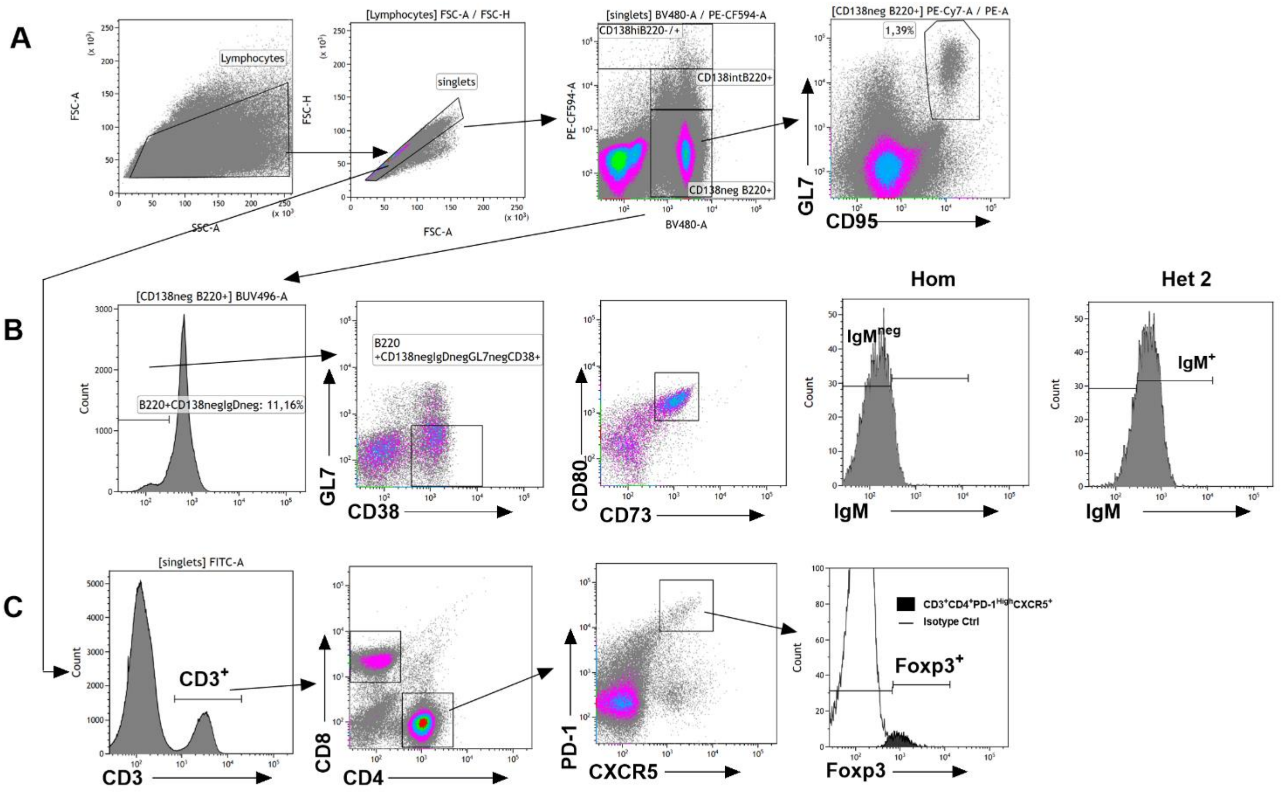

Supplementary Figure 1. Gating strategy. (A) Pre-plasmablast/plasmablast (B220^{+}CD138^{int}), plasmablast/PC (B220$^{+/-}$CD138^{high}) and GC B cells (B220^{+}CD138^{neg}GL7^{+}CD95^{+}). (B) Memory B cells (B220^{+}CD138^{neg}IgD^{neg}GL7^{neg}CD38^{+}), GC-derived memory B cells (B220^{+}CD138^{neg}IgD^{neg}GL7^{neg}CD38^{+} CD73^{+} CD80^{+}), IgM^{+} or IgM^{neg} GC-derived memory B cells. (C) T_{FH} and T_{FR} cells (CD3^{+}CD4^{+}CD8^{neg}PD-1^{+}CXCR5^{+} along with Foxp3^{neg} or Foxp3^{+} for T_{FH} or T_{FR}, respectively).

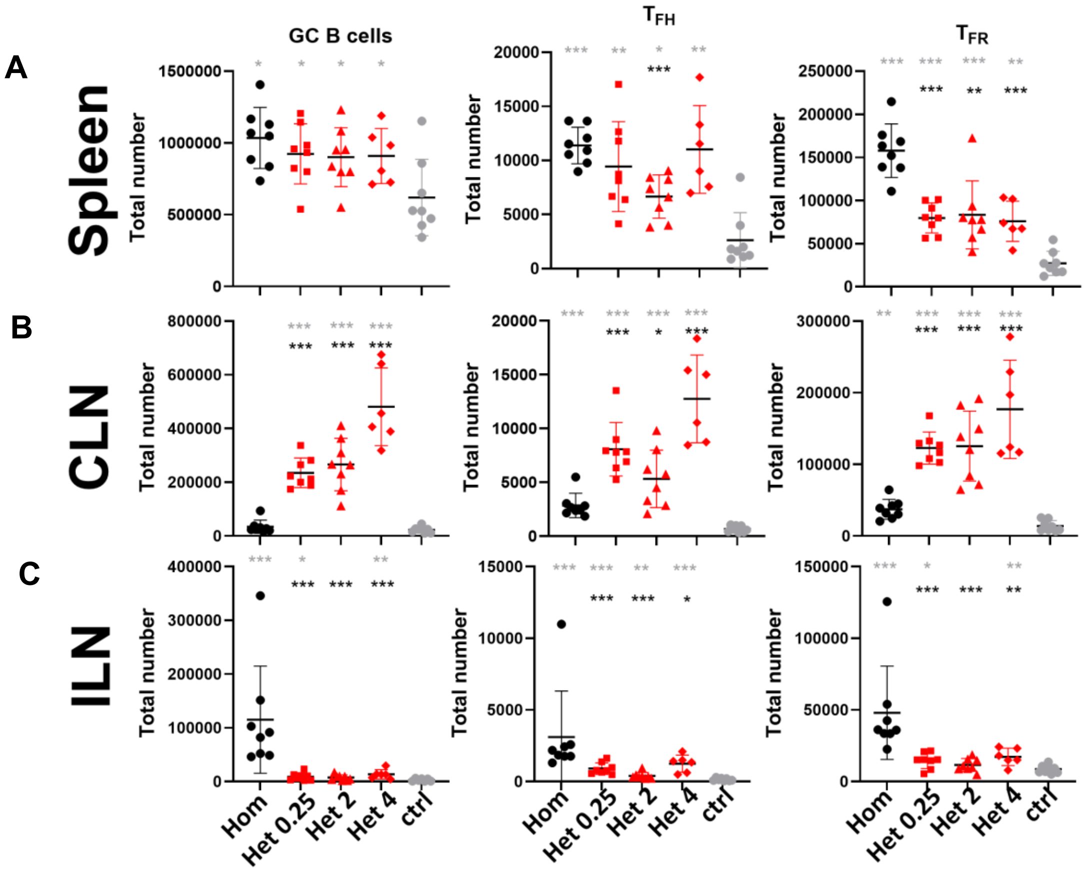

Supplementary Figure 2. Total number of GC B cells, T_{FH} and T_{FR} 14 days post-booster in (A) spleen, (B) CLNs, and (C) ILNs. Mice were immunized by different immunization schedules utilizing 0.25, 2 or 4 µg of Pn1-CRM_{197}, CAF01, and 2 µg of mmCT. Each symbol represents one mouse and results are shown as means ± SD in 6-8 mice per group. For statistical evaluation, Mann-Whitney U-test was used. Black stars represent p values after comparison of homologous s.c./s.c. group to heterologous s.c.i.n. groups and grey stars represent comparisons of all the groups to the control group. *$p \leq 0.05$, **$p \leq 0.01$, ***$p \leq 0.001$. Hom (Homologous s.c./s.c.) black circles, Het 0.25 (Heterologus s.c./i.n. 0.25 µg of Pn1-CRM197) red boxes, Het 2 (Heterologus s.c./i.n. 2 µg of Pn1-CRM_{197}) red triangles, Het 4 (Heterologus s.c./ i.n. 4 µg of Pn1-CRM_{197}) red rhombus and ctrl (control) grey circles.

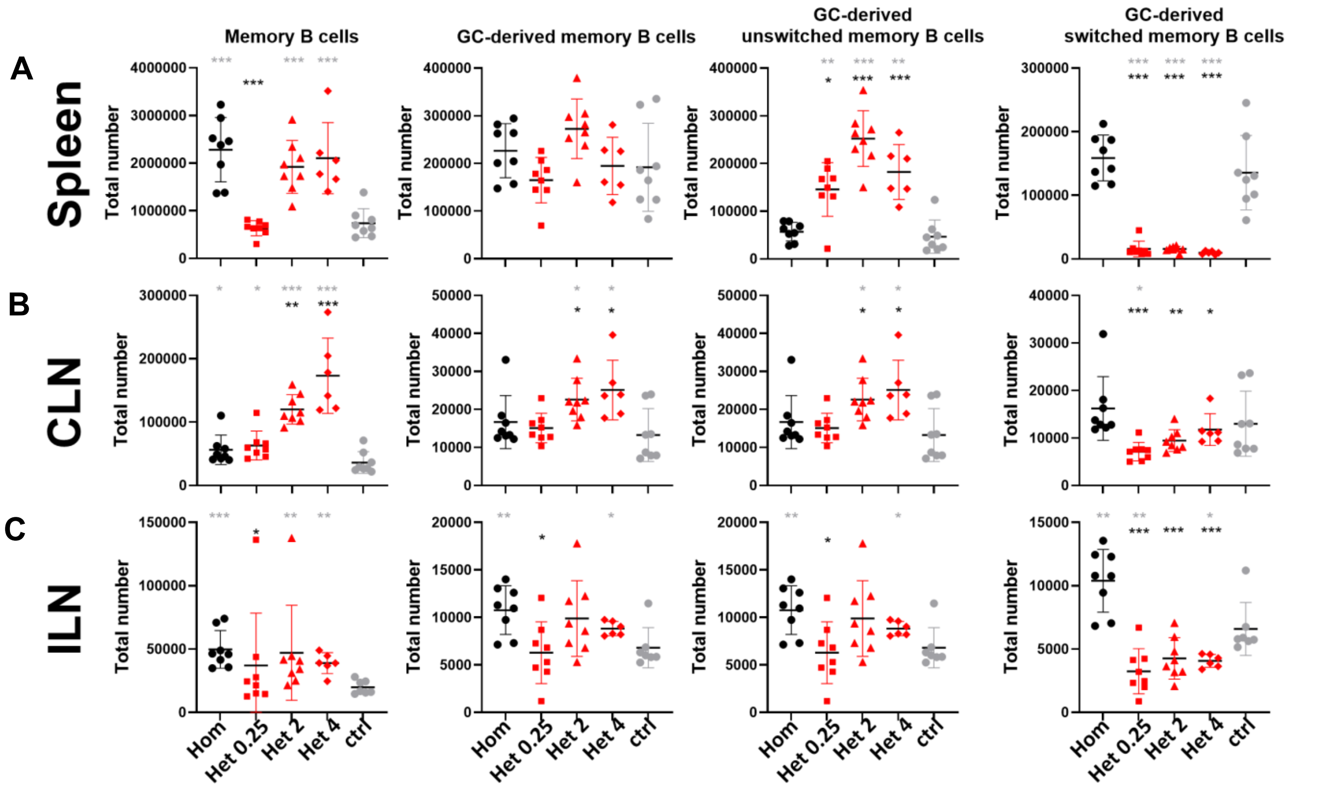

Supplementary Figure 3. Total number of memory B cells, GC-derived memory B cells, unswitched and switched GC-derived memory B cells in (A) spleen, (B) CLNs and (C) ILNs. Mice were immunized by different immunization schedules utilizing 0.25, 2 or 4 µg of Pn1-CRM$_{197}$, CAF01, and 2 µg of mmCT. Each symbol represents one mouse and results are shown as means ± SD in 6-8 mice per group. For statistical evaluation, Mann–Whitney U-test was used. Black stars represent p values after comparison of homologous s.c./s.c. group to heterologous i.n./i.n. groups and grey stars represent comparisons of all the groups to the control group. $^*p \leq 0.05$, $^{**}p \leq 0.01$, $^{***}p \leq 0.001$. Hom (Homologous s.c./s.c.) black circles, Het 0.25 (Heterologus s.c./ i.n. 0.25 µg of Pn1-CRM197) red boxes, Het 2 (Heterologus s.c./i.n. 2 µg of Pn1-CRM197) red triangles, Het 4 (Heterologus s.c./i.n. 4 µg of Pn1-CRM197) red rhombus and ctrl (control) grey circles.

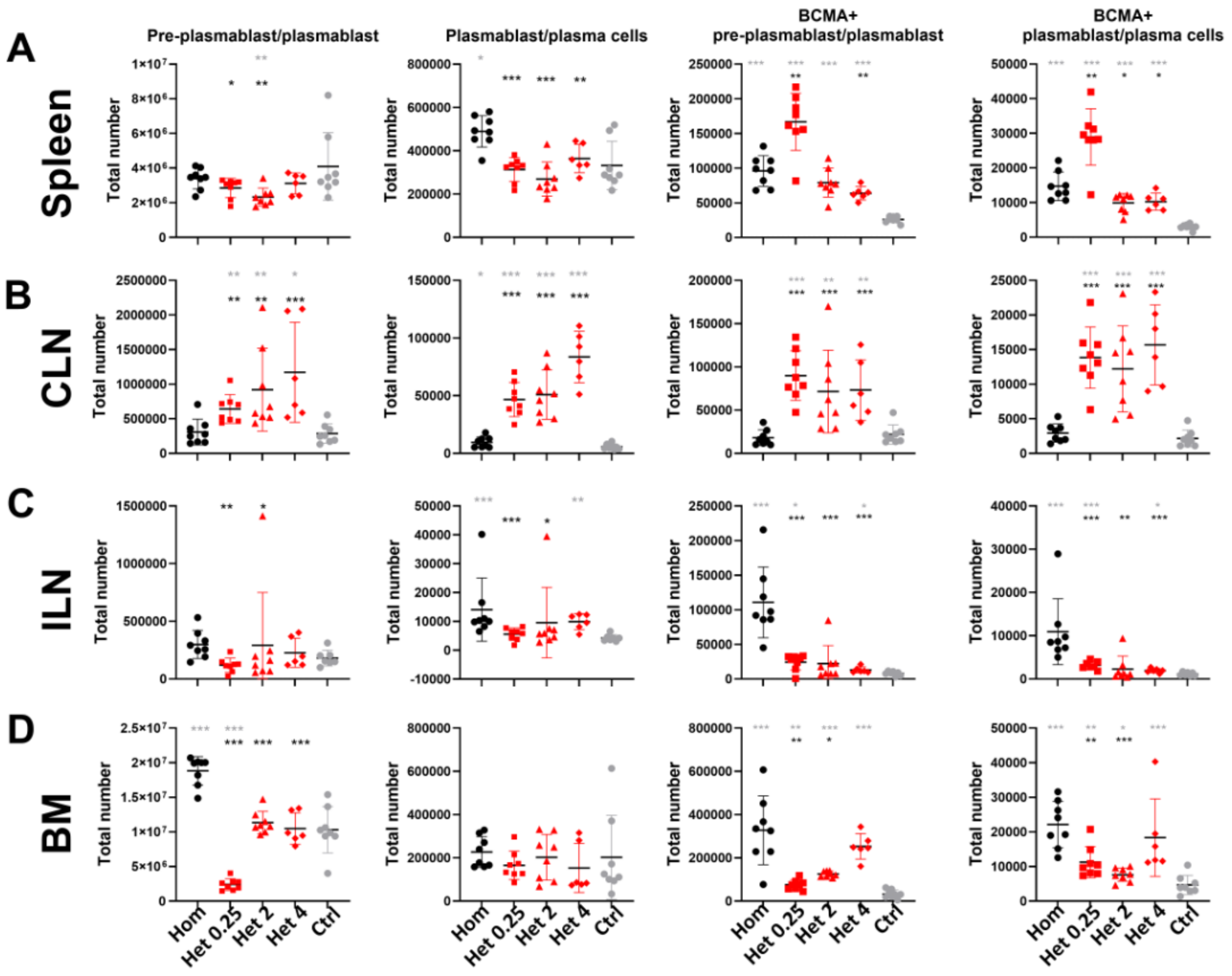

Supplementary Figure 4. Total number of pre-plasmablast/plasmablast, plasmablast/plasma cells and BCMA expression 14 days post-booster in (A) spleen, (B) CLNs, (C) ILNs and (D) BM. Mice were immunized by different immunization schedules utilizing 0.25, 2 or 4 µg of Pn1-CRM197, CAF01, and 2 µg of mmCT. Each symbol represents one mouse and results are shown as means ± SD in 6-8 mice per group. For statistical evaluation, Mann-Whitney U-test was used. Black stars represent p values after comparison of homologous s.c./s.c. group to heterologous i.n./i.n. groups and grey stars represent comparisons of all the groups to the control group. $*p \leq 0.05$, $**p \leq 0.01$, $***p \leq 0.001$. Hom (Homologous s.c./s.c.) black circles, Het 0.25 (Heterologus s.c./ i.n. 0.25 µg of Pn1-CRM197) red boxes, Het 2 (Heterologus s.c./i.n. 2 µg of Pn1-CRM197) red triangles, Het 4 (Heterologus s.c./ i.n. 4 µg of Pn1-CRM197) red rhombus and ctrl (control) grey circles.

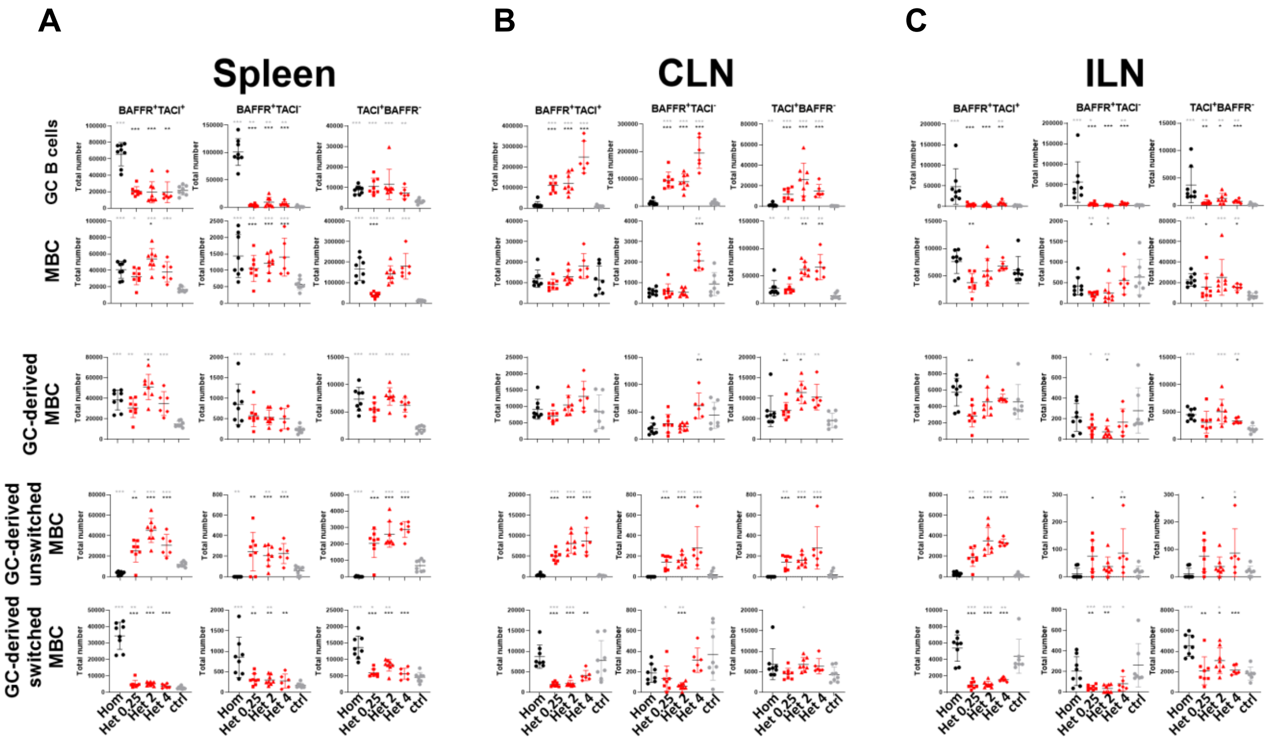

Supplementary Figure 5. Total number of BAFF-R and TACI expression on GC B cells, memory B cells, GC-derived memory B cells, unswitched and switched GC-derived memory B cells 14 days post-booster in (A) spleen, (B) CLNs, and (C) ILNs. Mice were immunized by different immunization schedules utilizing 0.25, 2 or 4 µg of Pn1-CRM197, CAF01, and 2 µg of mmCT. Each symbol represents one mouse and results are shown as means ± SD in 6-8 mice per group. For statistical evaluation, Mann-Whitney U-test was used. Black stars represent p values after comparison of homologous s.c./s.c. group to heterologous i.n./i.n. groups and grey stars represent comparisons of all the groups to the control group. *$p \leq 0.05$, **$p \leq 0.01$, ***$p \leq 0.001$. Hom (Homologous s.c./s.c.) black circles, Het 0.25 (Heterologus s.c./ i.n. 0.25 µg of Pn1-CRM197) red boxes, Het 2 (Heterologus s.c./ i.n. 2 µg of Pn1-CRM197) red triangles, Het 4 (Heterologus s.c./ i.n. 4 µg of Pn1-CRM197) red rhombus and ctrl (control) grey circles.

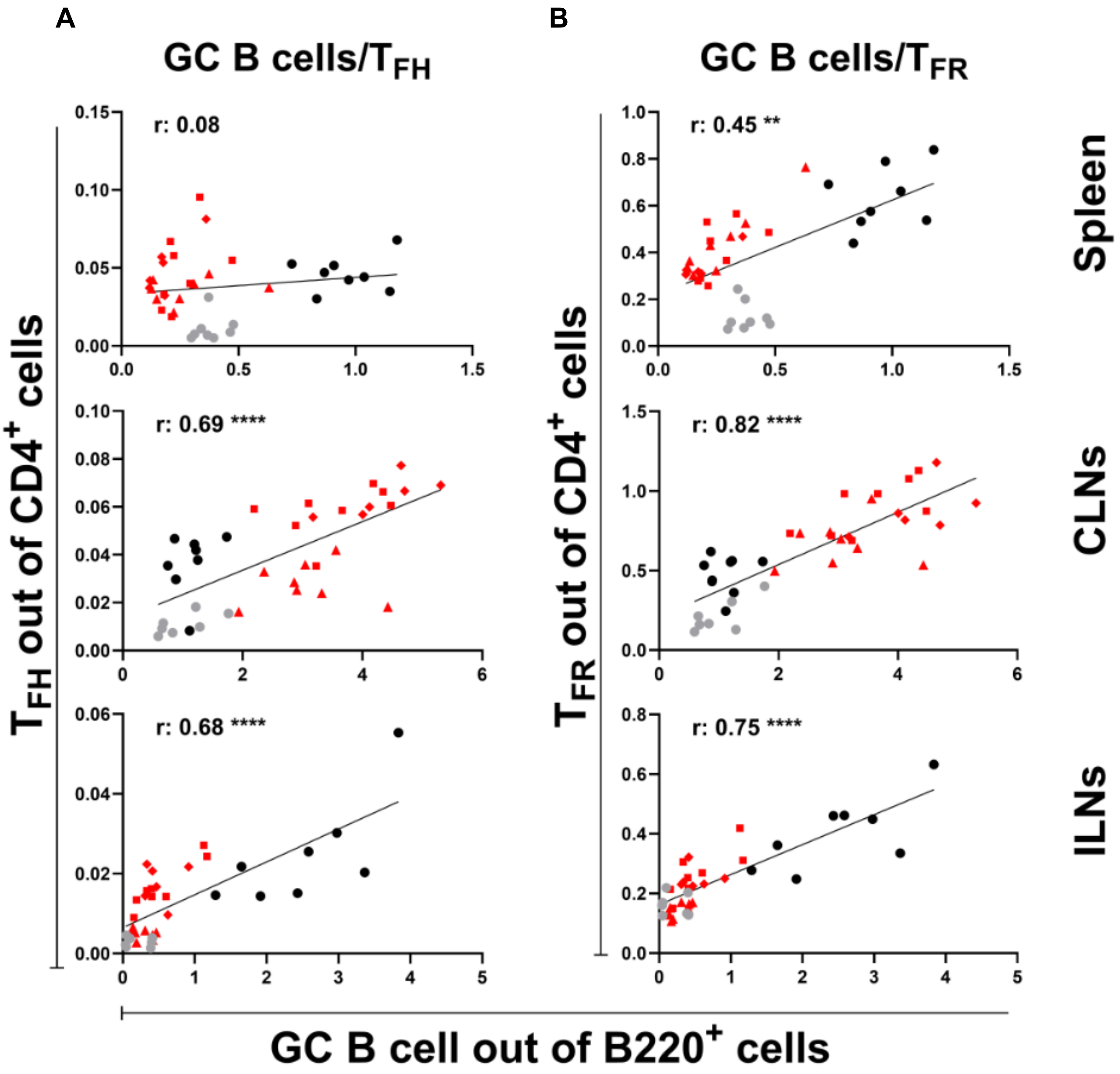

Supplementary Figure 6. Correlation of T$_{FH}$ and T$_{FR}$ with GC B cells. (A) GC B cells and T$_{FH}$ correlations in spleen, CLNs and ILNs 14 days post-booster. (B) GC B cells and T$_{FR}$ correlations in spleen, CLNs and ILNs 14 days post-booster. Mice were immunized by different immunization schedules utilizing 0.25, 2 or 4 µg of Pn1-CRM197, CAF01, and 2 µg of mmCT. Each symbol represents one mouse and results are shown for 6-8 mice per group. For statistical evaluation, Spearman correlation was used. *p ≤ 0.05, **p ≤ 0.01, ***p ≤ 0.001. Hom (Homologous s.c./s.c.) black circles, Het 0.25 (Heterologus s.c./ i.n. 0.25 µg of Pn1-CRM197) red boxes, Het 2 (Heterologus s.c./ i.n. 2 µg of Pn1-CRM197) red triangles, Het 4 (Heterologus s.c./ i.n. 4 µg of Pn1-CRM197) red rhombus and ctrl (control) grey circles.

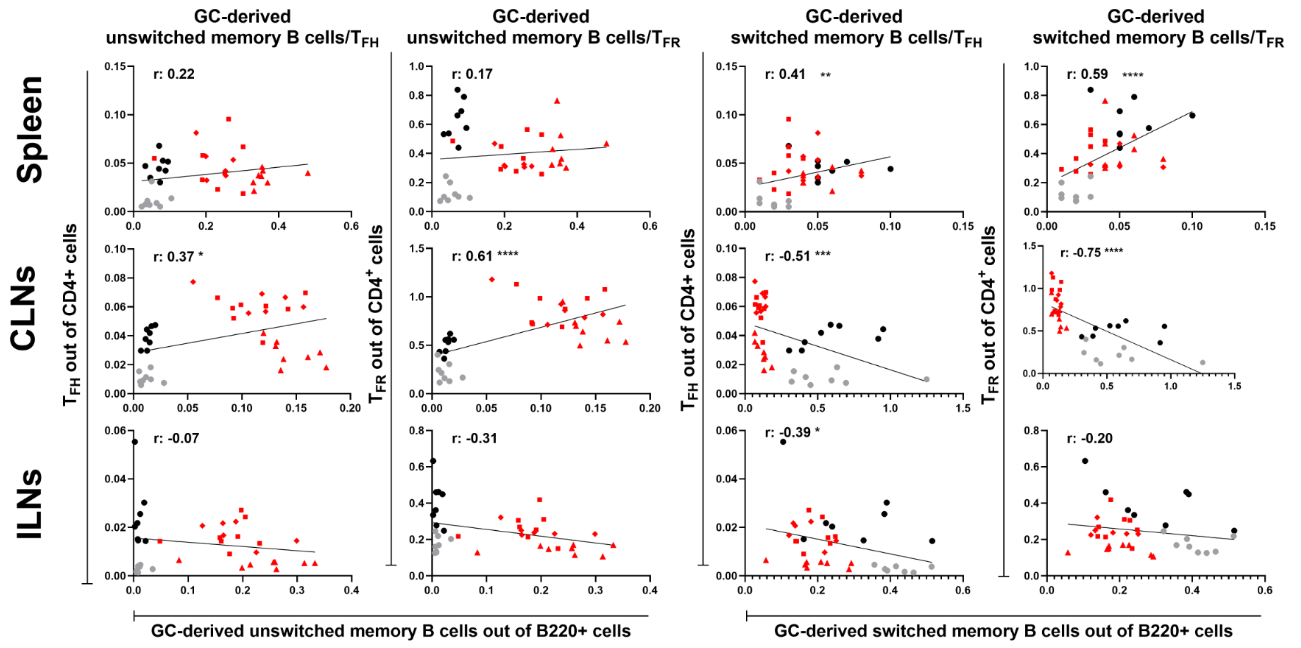

Supplementary Figure 7. Correlation of T_{FH} and T_{FR} with GC-derived unswitched and switched memory B cells. (A) Correlation of T_{FH} and T_{FR} with GC-derived unswitched and switched memory B cells in spleen 14 days post-booster. (B) Correlation of T_{FH} and T_{FR} with GC-derived unswitched and switched memory B cells in CLNs 14 days post-booster. (C) Correlation of T_{FH} and T_{FR} with GCderived unswitched and switched memory B cells in ILNs 14 days post-booster. Mice were immunized by different immunization schedules utilizing 0.25, 2 or 4 µg of Pn1-CRM197, CAF01, and 2 µg of mmCT. Each symbol represents one mouse and results are shown for 6-8 mice per group. For statistical evaluation, Spearman correlation was used. $*p \leq 0.05$, $**p \leq 0.01$, $***p \leq 0.001$. Hom(Homologous s.c./s.c.) black circles, Het 0.25 (Heterologus s.c./ i.n. 0.25 µg of Pn1-CRM197) red boxes, Het 2 (Heterologus s.c./ i.n. 2 µg of Pn1-CRM197) red triangles, Het 4 (Heterologus s.c./ i.n. 4 µg of Pn1-CRM197) red rhombus and ctrl (control) grey circles.

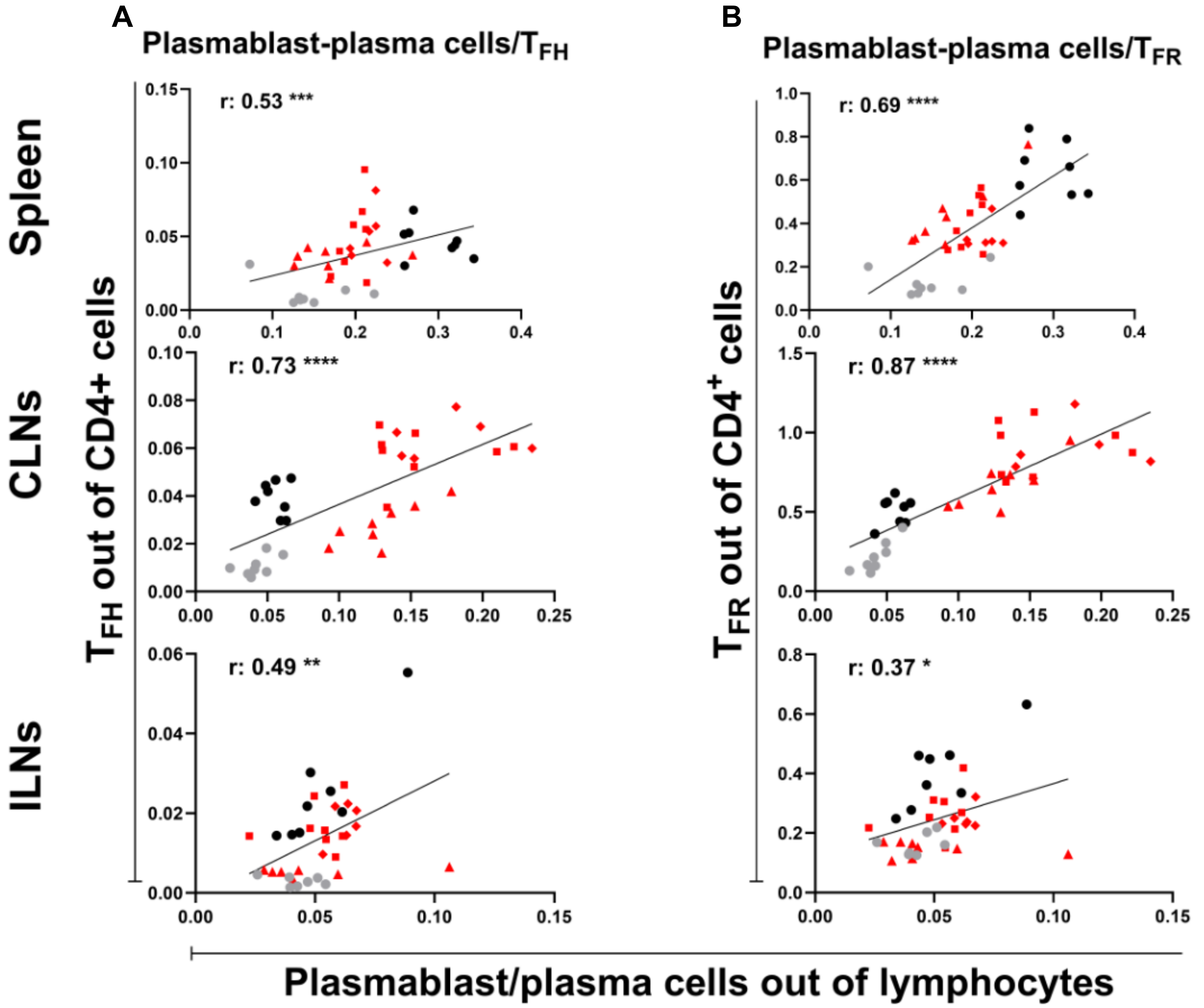

Supplementary Figure 8. Correlation of T_{FH} and T_{FR} with plasmablast/plasma cells. (A) Correlation of T_{FH} with plasmablast/PC in spleen, CLNs and ILNs 14 days post-booster. (B) Correlation of T_{FR} with plasmablast/PC in spleen, CLNs and ILNs 14 days post-booster. Mice were immunized by different immunization schedules utilizing 0.25, 2 or 4 µg of Pn1-CRM197, CAF01, and 2 µg of mmCT. Each symbol represents one mouse and results are shown for 6-8 mice per group. For statistical evaluation, Spearman correlation was used. *p ≤ 0.05, **p ≤ 0.01, ***p ≤ 0.001. Hom (Homologous s.c./s.c.) black circles, Het 0.25 (Heterologus s.c./ i.n. 0.25 µg of Pn1-CRM197) red boxes, Het 2 (Heterologus s.c./ i.n. 2 µg of Pn1-CRM197) red triangles, Het 4 (Heterologus s.c./ i.n. 4 µg of Pn1-CRM197) red rhombus and ctrl (control) grey circles.
